# Supplementary material for: Orphan receptor GPR153 facilitates vascular damage responses by modulating cAMP levels, YAP/TAZ signaling, and NF-κB activation
Source: Nat Commun. 2025 Jul 7;16:6232. doi: 10.1038/s41467-025-61057-w (PMC12234975; doi:10.1038/s41467-025-61057-w)
Supplement: Supplementary file 1 — Supplementary Information [file 41467_2025_61057_MOESM1_ESM.pdf]

Supplemental Figures for Shao et al., 2025:
Orphan receptor GPR153 facilitates vascular damage responses by modulating cAMP levels, YAP/TAZ signaling, and NF-κB activation

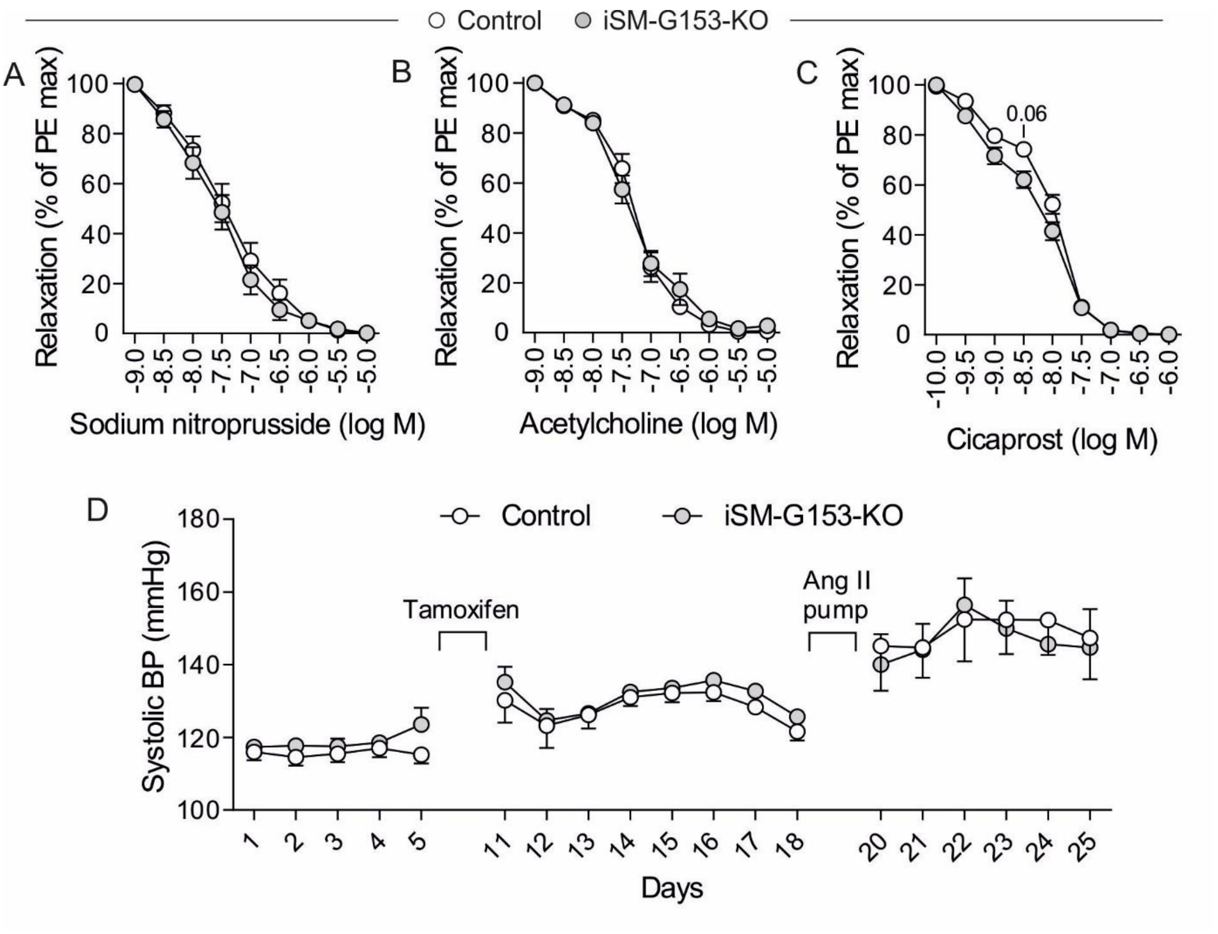

Supplemental Figure 1: Contractile responses and blood pressure regulation in iSM-G153-KOs. A-C, Dose-response curves for different vasodilators were determined by wire myography in mesenteric arteries from control mice and iSM-G153-KOs (responses measured after precontraction with 10 µM phenylephrine (PE) and expressed as percentage of maximal PE contraction; n=12-14 (1-2 vessels per mouse). **D,** Telemetric recording of systolic blood pressures in control and iSM-G153-KO mice before and after tamoxifen induction and subsequent implantation of Ang II-releasing miniosmotic pumps (days 4-10 and 19 not recorded).

Data are means ± SEM; comparisons between genotypes were performed using two-way ANOVA and Sidak's multiple comparisons test (A-C) or two-way repeated measures ANOVA and Bonferroni's post-hoc test (D).

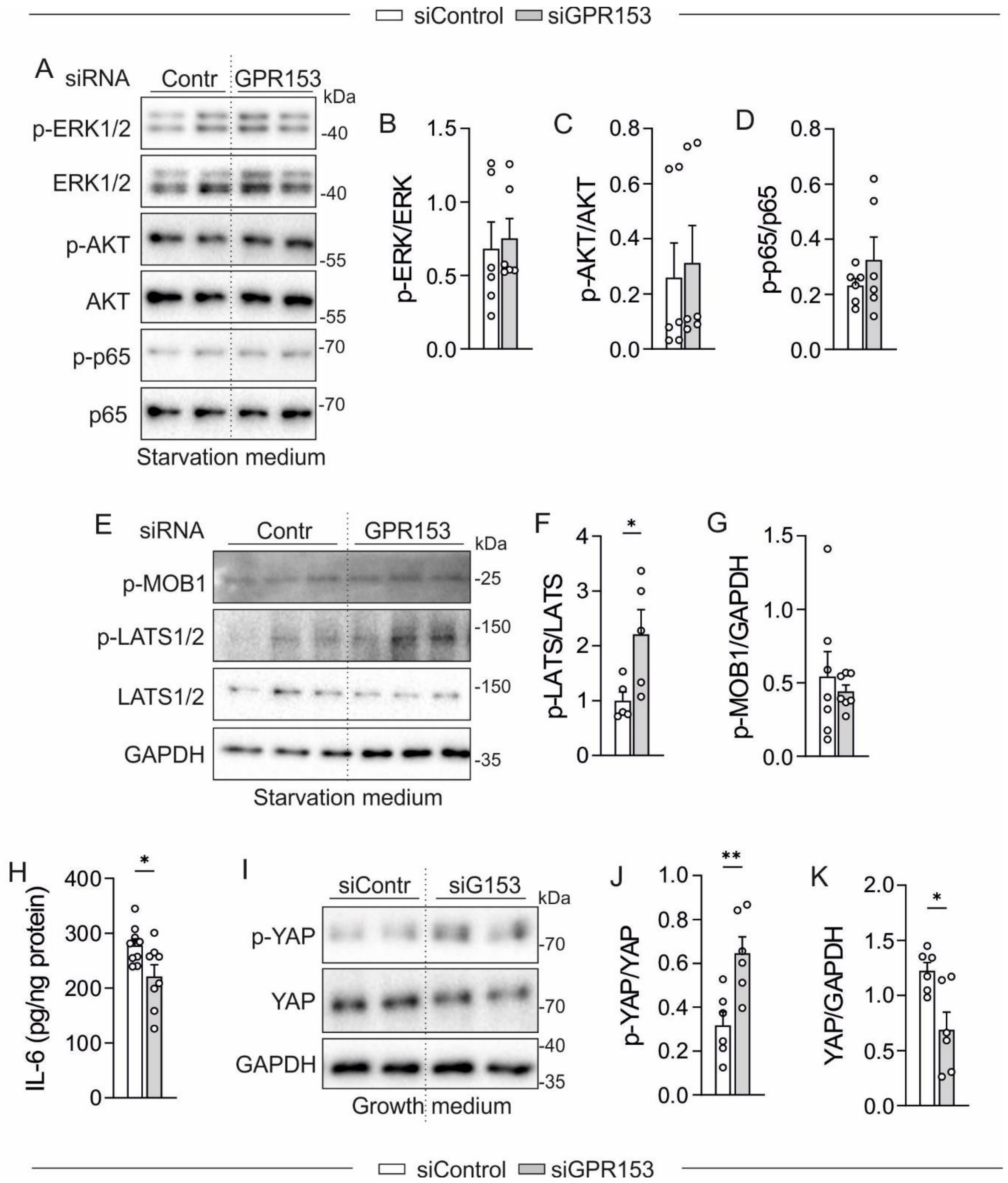

Supplemental Figure 2: Effect of GPR153 deficiency on cellular signaling cascades in hCASMCs. **A-G,** Control and GPR153 knockdown hCASMCs were grown in the absence of serum and growth factors for 48 h, then analyzed by immunoblotting (GAPDH as loading control, n=5-7). **H,** IL-6 levels in the supernatant of growth medium-cultured control and GPR153 knockdown hCASMCs were determined by ELISA (n=8-9). **I-K,** Control and GPR153 knockdown hCASMCs were starved for 48 hours and then exposed for 15 min to growth medium (5% FBS, 0.1% insulin, 0.2% basic human fibroblast growth factor, 0.1% human epidermal growth factor), then analyzed by immunoblotting (GAPDH as loading control, n=6).

Data are means ± SEM; differences between genotypes were analyzed using unpaired two-sided t test. *, $P < 0.05$; **, $P < 0.01$.

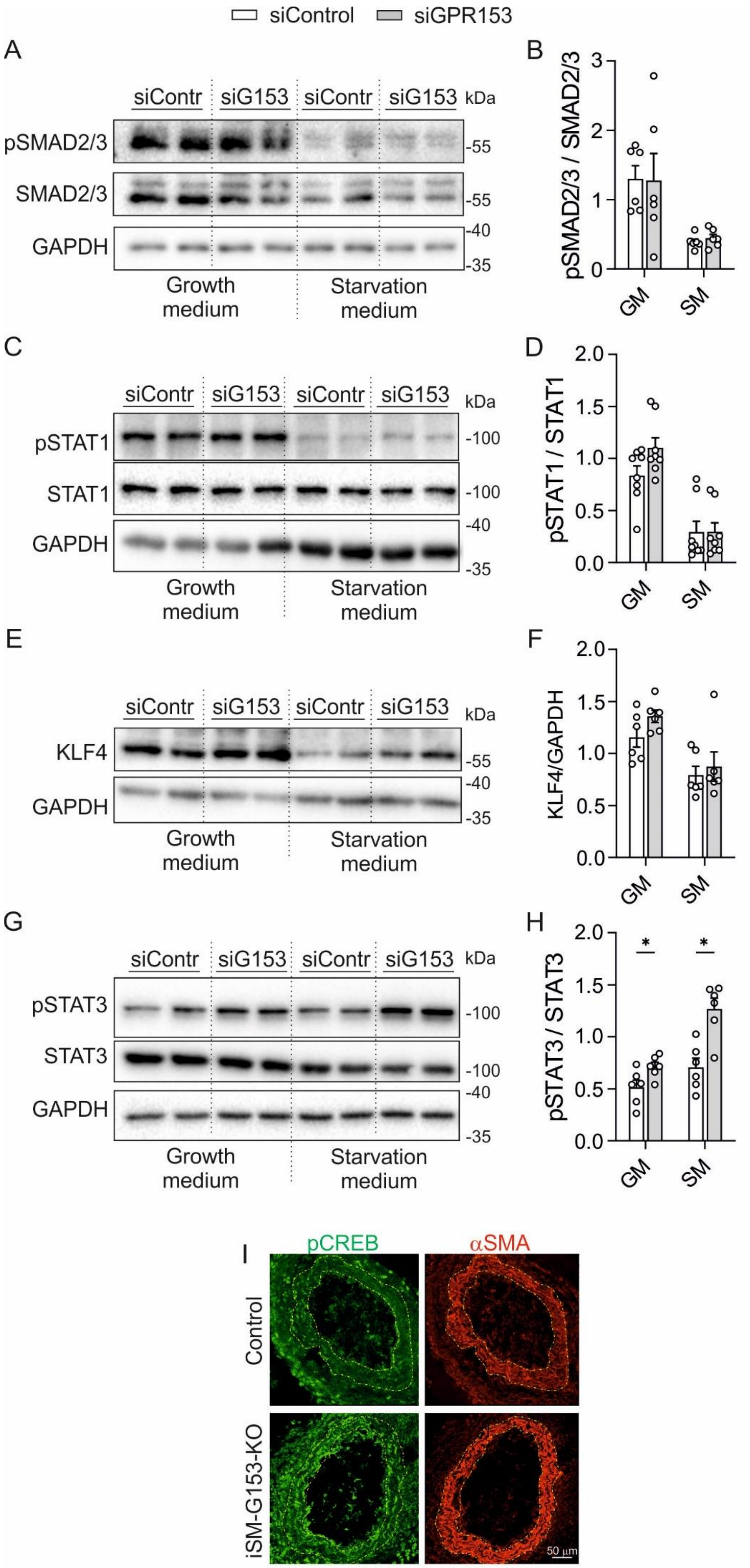
siControl
siGPR153
A
siContr
siG153
siContr
siG153
kDa
pSMAD2/3
SMAD2/3
GAPDH
-55
-55
-40
-35
Growth medium
Starvation medium
B
pSMAD2/3 / SMAD2/3
GM
SM
C
pSTAT1
STAT1
GAPDH
-100
-100
-40
-35
D
pSTAT1 / STAT1
E
KLF4
GAPDH
F
KLF4/GAPDH
G
pSTAT3
STAT3
GAPDH
H
pSTAT3 / STAT3
*
*
I
pCREB
αSMA
Control
iSM-G153-KO
50 µm

Supplemental Figure 3: Effect of GPR153 deficiency on signaling cascades in human and murine SMCs. A-H, hCASMCs transfected with control siRNA (siContr) or GPR153-specific siRNA (siG153) were grown for 48 h in the presence of serum- / growth factor-containing medium (growth medium, GM) or in growth factor-reduced medium (starvation medium, SM), then analyzed by immunoblotting with the indicated antibodies (GAPDH as loading control, n=6-8). **I,** Exemplary microphotographs for manuscript Figure 5W: Carotid arteries harvested on day 7 after ligation from control and iSM-G153-KOs were cryosectioned and immunostained with antibodies directed against αSMA and pCREB: hatched yellow lines indicate media borders as judged by αSMA staining.

Data are means ± SEM; differences between genotypes were analyzed using two-way ANOVA and Sidak's post-hoc test; *, $P < 0.05$.

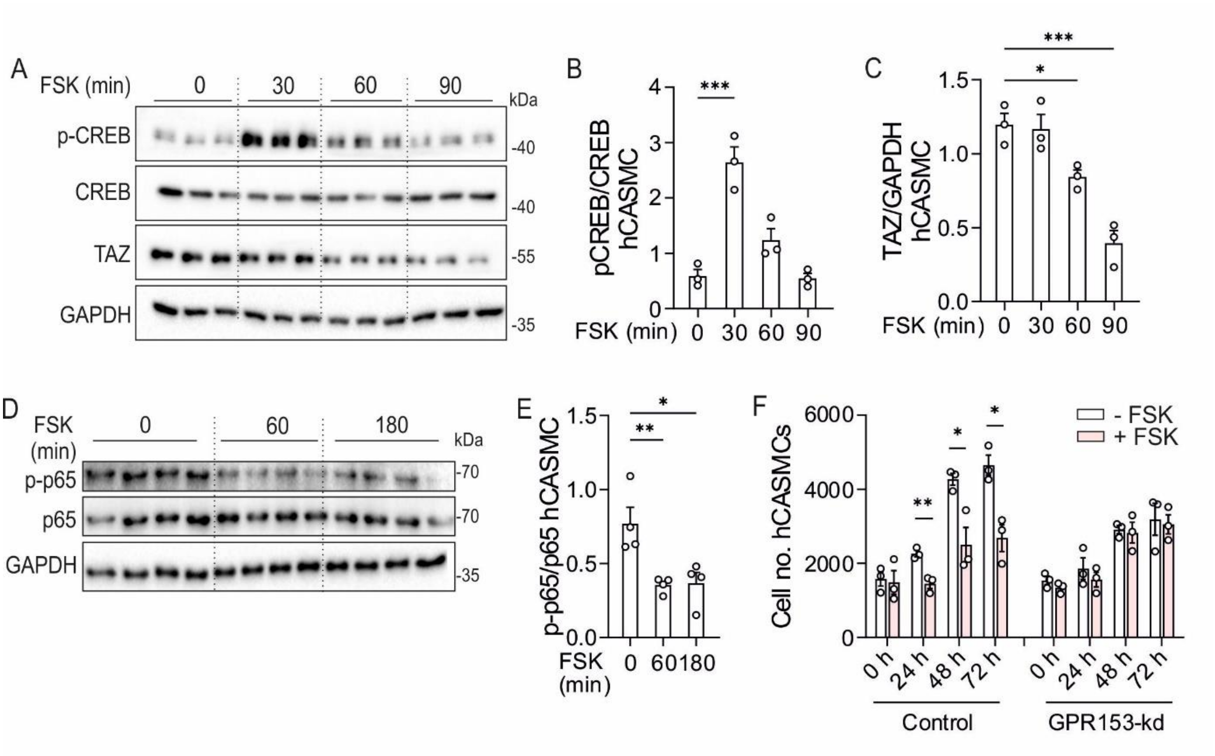

Supplemental Figure 4: GPR153 influences FSK-induced signaling and proliferation in hCASMCs. A-E, Immunoblotting of from hCASMC lysates in the basal state or after stimulation with 1 µM FSK for the indicated times: representative blots (A,D, GAPDH as loading control) and densitometric analysis of signal strength (B,C,E). **F,** Effect of 1 µM FSK on cell number expansion in control and GPR153 knockdown hCASMCs (n=3).

Data are means ± SEM; differences were analyzed using one-way ANOVA with Dunnett's multiple comparisons test (B,C,E) or unpaired two-sided t tests corrected for multiple testing with the two-stage step-up method (Benjamini, Krieger, and Yekutieli) (F). *, $P < 0.05$; **, $P < 0.01$; ***, $P < 0.001$.

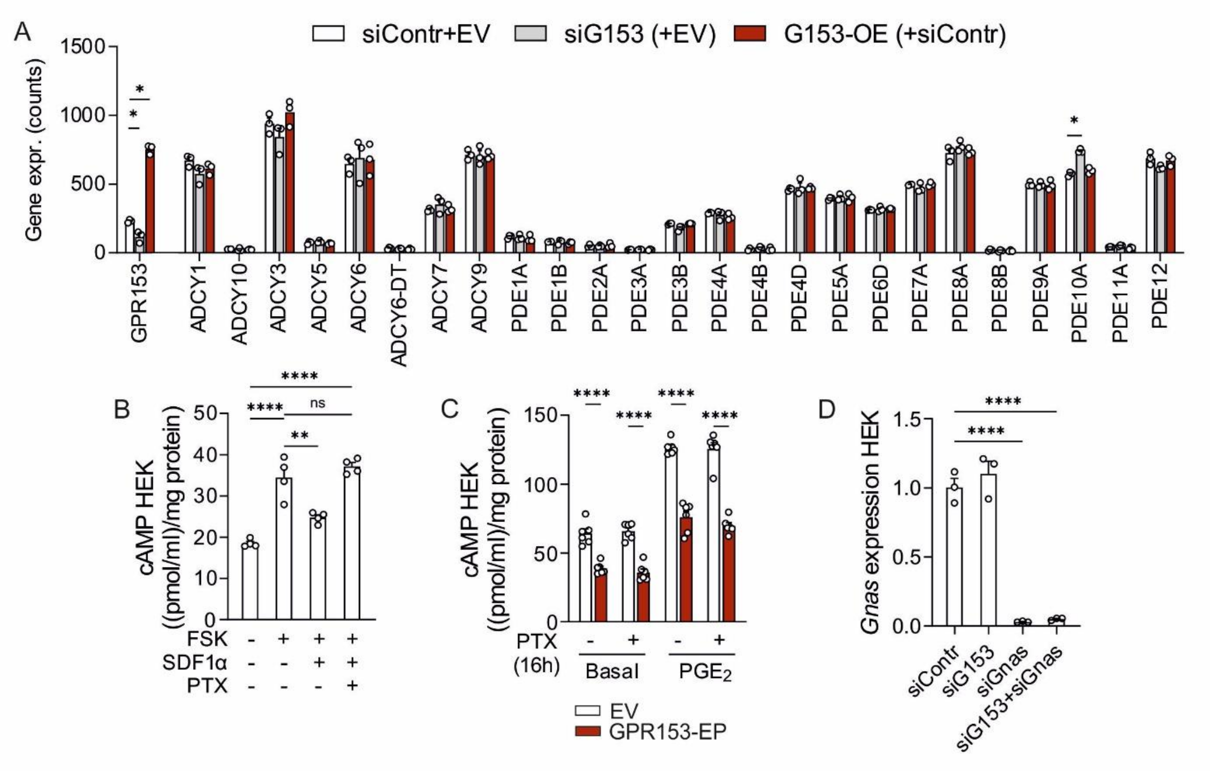

Supplemental Figure 5: GPR153 function in HEK cells. A, Library size-normalized counts detected by RNA sequencing in HEK293T cells transfected with scrambled control siRNA and empty vector (siContr+EV), siRNA directed against GPR153 and empty vector (siG153 (+EV)) or control siRNA and GPR153 expression plasmid (G153-OE (+siContr)). **B,** Effect of PTX (100 ng/ml, 3h) on SDF1α-mediated inhibition of FSK-induced cAMP production in CXCR4-overexpressing HEK cells (n=4, SDF1α and FSK both 1 μM, 10 min). **C,** Effect of PTX (100 ng/ml, 16h) on PGE_2 (1 μM, 30 min)-induced cAMP production was determined by ELISA in EV- and GPR153-EP-transfected HEK cells (n=6). **D,** *Gnas* expression in HEK cells transfected with control siRNA (SiContr) or siRNA directed against *Gnas* (n=3).

Data are means ± SEM; comparisons between genotypes were performed using multiple unpaired two-tailed t test (Benjamini Hochberg correction) (A), one-way ANOVA with Sidak's multiple comparisons test (B,D), or two-way ANOVA and Sidak's post-hoc test (C). *, $P < 0.05$; **, $P < 0.01$; ****, $P < 0.0001$.

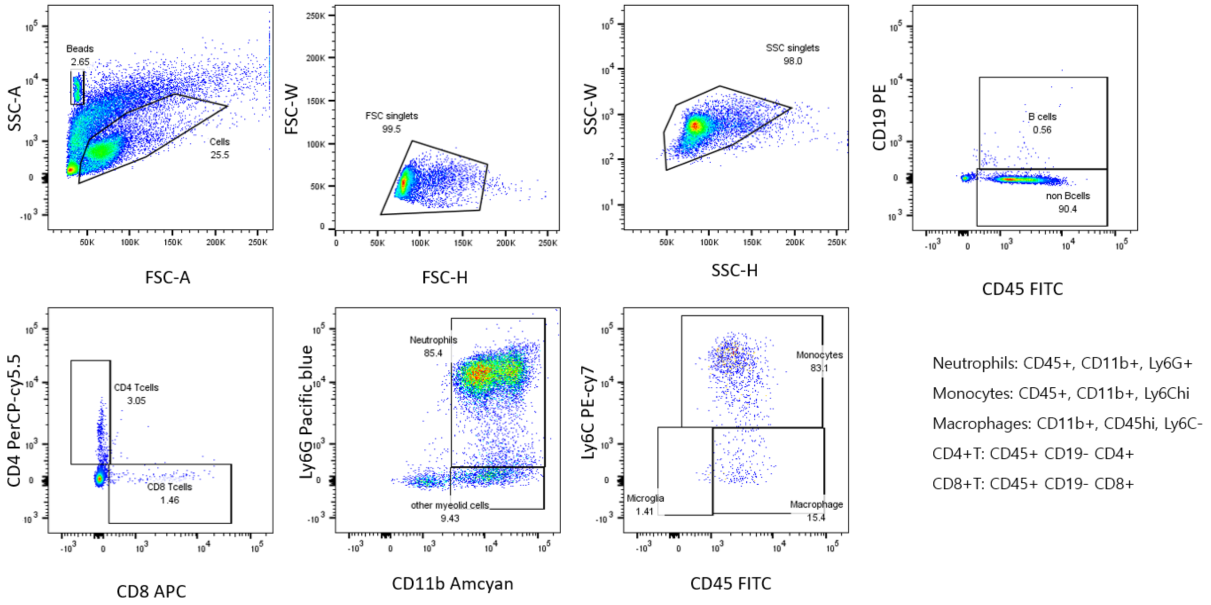

Supplemental Figure 6: Gating strategy for the analysis of spinal cord leukocyte infiltration in the EAE model (pertaining to Figure 8I).
